# Improvement of the Rice “Easy-to-Shatter” Trait via CRISPR/Cas9-Mediated Mutagenesis of the *qSH1* Gene

**DOI:** 10.3389/fpls.2020.00619

**Published:** 2020-05-25

**Authors:** Xiabing Sheng, Zhizhong Sun, Xuefeng Wang, Yanning Tan, Dong Yu, Guilong Yuan, Dingyang Yuan, Meijuan Duan

**Affiliations:** ^1^College of Bioscience and Biotechnology, Hunan Agricultural University, Changsha, China; ^2^State Key Laboratory of Hybrid Rice, Hunan Hybrid Rice Research Center, Changsha, China; ^3^Hunan Academy of Agricultural Sciences, Changsha, China

**Keywords:** CRISPR/Cas9, heterosis utilization, *qSH1*, rice, seed shattering

## Abstract

“Easy-to-shatter” trait is a major cause of rice crop yield losses, emphasizing the economic value of developing elite rice cultivars with reduced seed shattering capable of achieving higher yields. In the present study, we describe the development of new *indica* rice lines that exhibit lower rates of seed shattering following the targeted CRISPR/Cas9-mediated editing of the *qSH1* gene. We were able to identify *qSH1* mutant T0 transgenic plants, with transgene-free homozygous mutants being obtained via segregation in the T1 generation. We then utilized two T2 transgene-free homozygous lines in order to assess the degree of seed shattering and major agronomic traits of these mutant lines and of wild-type rice plants (HR1128-WT). This approach revealed that *qsh1* homozygous mutant lines exhibited significantly reduced seed shattering relative to HR1128-WT without any significant changes in other analyzed agronomic traits. We then used these mutant lines to develop new promising hybrid rice lines with intermediate seed shattering. Overall our results reveal that combining targeted gene editing via CRISPR/Cas9 with heterosis utilization approach can allow for the efficient development of novel promising hybrid rice cultivars that exhibit a intermediate of seed shattering, thereby ensuring better stability and improved rice yields.

## Introduction

Rice (*Oryza sativa* L.) is a staple crop and one of the most important food sources in the world, being consumed by over half of the global population (Wang et al., 2013). In the wild, seed shattering is an advantageous behavior for rice. However, in a cultivated context, domesticated rice varieties that exhibit the “easy-to-shatter” trait can result in serious yield losses during the harvesting process (Doebley, 2006; Fuller et al., 2009). As such, there have been many efforts to breed rice varieties with an intermediate seed shattering phenotype in an effort to stabilize rice yields. A number of rice quantitative trait loci (QTLs) and genes associated with seed shattering have been cloned to date, such as *SH-h*, *SH3*, *SH4*, *qSH1*, *SHAT1*, and *SSH1* (Li et al., 2006; Konishi et al., 2006; Lin et al., 2007; Ji et al., 2010; Lv et al., 2018; Jiang et al., 2019). Few of these genes, however, have been leveraged to develop elite rice varieties, particularly hybrid rice varieties. The *qSH1* gene is an important QTL located on chromosome1 that is associated with seed shattering and that codes for a BEL1-type homeobox-containing protein. Loss of *qSH1* expression in the abscission layer results in a significantly improved strong seed shattering phenotype (Konishi et al., 2006).

Hybrid rice plants offer yields that are 10–20% higher than those of conventional rice, and as a result these hybrid cultivars have been cultivated in over 40 countries throughout the world (Su et al., 2012; Zhou et al., 2016). At present, *indica* hybrids are the dominant crops used for commercial rice production, particularly in southern China. Such *indica* varietals, however, exhibit a greater propensity for the “easy-to-shatter” trait than do *japanic* rice cultivars. Previous reports suggest that certain hybrid rice varieties can suffer a 5.8–8.6% harvest yield loss owing to their susceptibility to seed shattering (Ye et al., 2012). As such, the development of genetically modified rice cultivars with an intermediate seed shattering phenotype has been a priority in recent years. Conventional breeding strategies require 5–10 years in order to develop novel seed shattering cultivars via mutagenesis and subsequent crossing and backcrossing. The current lack of robust germplasm resources associated with weaker seed shattering and the inefficient nature of such conventional breeding strategies have significantly constrained efforts to produce intermediate seed shattering rice cultivars to date. It is therefore essential that novel technologies be leveraged in an effort to reduce the propensity of rice plants to readily undergo seed shattering.

Many recent advances in plant genomic editing have been made through the use of tools such as sequence-specific nucleases (SSNs), including zinc finger nucleases (ZFNs), transcription activator-like effector nucleases (TALENs), and clustered regularly interspaced short palindromic repeats (CRISPR)/CRISPR-associated (Cas) 9 (CRISPR/Cas9) (Townsend et al., 2009; Li et al., 2012; Feng et al., 2013; Nekrasov et al., 2013; Baltes and Voytas, 2015; Hyun et al., 2015; Svitashev et al., 2015; Weeks et al., 2016). Of these tools, CRISPR/Cas9-mediated gene editing has been widely applied as a means of rapidly and reliably conducting genomic editing in important crops such as rice (Feng et al., 2013; Miao et al., 2013; Shan et al., 2013; Xu et al., 2014), maize (Liang et al., 2014; Feng et al., 2016; Zhu et al., 2016), tomato (Brooks et al., 2014; Ito et al., 2015), potato (Wang et al., 2015), wheat (Upadhyay et al., 2013; Shan et al., 2014; Wang et al., 2014), sorghum (Jiang et al., 2013), and soybean (Jacobs et al., 2015).

In the present study, we conducted the targeted mutagenesis of the *qSH1* gene via a CRISPR/Cas9-mediated approach. This allowed us to develop *qsh1* mutants on the HR1128 and Guangzhan63-4S (GZ63-4S) backgrounds, with both of these being parental lines of the commonly utilized the *indica* hybrid rice cultivar-GuangLiangYou1128 (GLY1128), which has many excellent agricultural traits, but with a strong seed shattering phenotype. Through gene editing approach, we were able to develop homozygous T1 mutant plants. These *qsh1* mutants exhibited significant reductions in seed shattering activity without any changes in other major agronomic traits relative to wild-type plants under normal growth conditions. Furthermore, the seed shattering propensities of the S1 and S2 promising hybrid rice lines were significantly reduced following crossing with our *qsh1* mutants with background of HR1128 and GZ63-4S.

## Materials and Methods

### Plant Materials and Growth

The HR-qsh1-ab, HR-qsh1-1, HR-qsh1-23, GZ-qsh1-ab, GZ-qsh1-1, and GZ-qsh1-23 mutants were produced via using the pYLCRISPR/Cas9-qsh1-Tab, T1, T23 vector to transform plants of the HR1128 and GZ63-4S rice varieties via *Agrobacterium*-mediated transformation. Plants were cultured at 28–35°C in a greenhouse in Changsha, or were grown in the transgenic crop planting field of the Hunan Hybrid Rice Research Centre in Changsha or Sanya during the standard rice-growing season.

### Vector Construction

**FIGURE 1:**
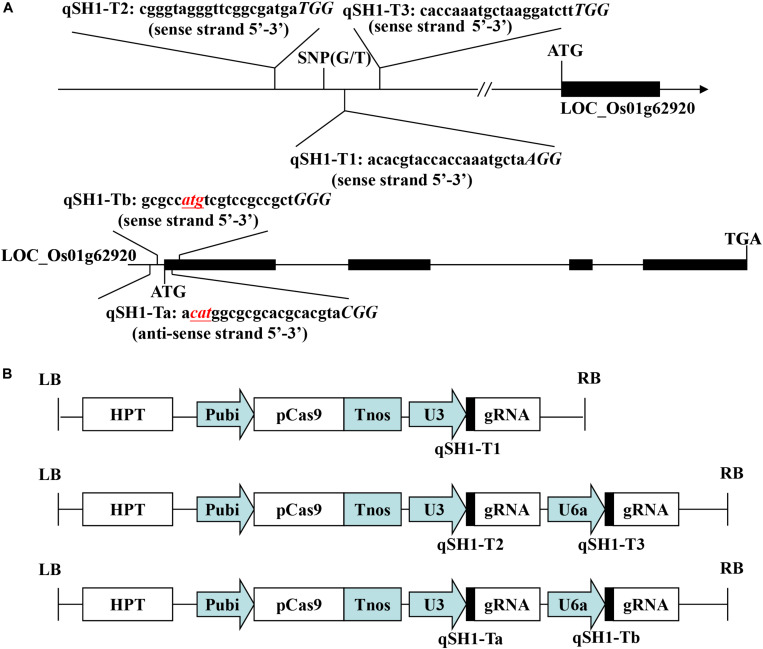
Target sites of the CRISPR/Cas9-qSH1-T1, T23, Tab vector. **(A)** Schematic of the *qSH1* gene structure and target site. Exons and introns are indicated with black rectangles and black lines, respectively. Both the translation initiation codon (ATG) and the termination codon (TGA) are shown. The target site nucleotides are shown in lowercase, and the protospacer adjacent motif (PAM) site is capitalized and italicized. **(B)** A schematic presentation of the T-DNA structure in the CRISPR/Cas9-mediated genome editing construct. The expression of Cas9 is driven by the maize ubiquitin promoter (Pubi); the expression of the sgRNA scaffold is driven by the rice U3 or U6a small nuclear RNA promoter (OsU3 or OsU6a); the expression of hygromycin (HPT) is driven by two CaMV35S promoter (2 × 35S); Tnos, gene terminator; LB and RB, left border and right border, respectively.

The pYLCRISPR/Cas9-qsh1-Tab, T1, T23 vector (pC-qsh1-Tab, T1, T23) was constructed as in previous reports (Ma et al., 2015). The gRNA expression vector (pYLgRNA-U3/U6a) and the Cas9 plant expression vector (pYLCRISPR/Cas9) were from Pro. Yao-guang Liu of the South China Agricultural University. For targeted mutagenesis of these rice plants, we selected a candidate target sequences composed of 19–20 bases upstream of the PAM motif (Figure 1A). We additionally obtained the target site sequence-containing primers qsh1-TaF/R, qsh1-TbF/R, qsh1-T1F/R, qsh1-T2F/R, and qsh1-T3F/R (Supplementary Table S1) from BLAST CRISPR-GE^1^ and NCBI^2^ as a means of ensuring that no off-target gene targeting occurred via this approach.

For pC-qsh1-Tab vector preparation, the target site qsh1-Ta and qsh1-Tb sequence-containing chimeric primers were cloned into the sgRNA expression cassettes pYLsgRNA-U3 and pYLsgRNA-U6a, respectively, at a *Bsa*I site (Ma et al., 2015). Synthesis of the gRNA expression cassette was achieved using the U-F/gRNA-R primers (Supplementary Table S1) for an initial round of overlapping PCR, followed by a second round using the site-specific B1’/B2 and B2’/BL primers (Supplementary Table S1). After this, the target sgRNA (qsh1-Ta-sgRNA and qsh1-Tb-sgRNA) expression cassettes were ligated into the pYLCRISPR/Cas9Pubi-H vector (Ma et al., 2015), yielding a pC-qsh1-Tab construct featuring a Cas9p expression cassette (Pubi:NLS:Cas9p:NLS:Tnos) and a hygromycin resistance cassette (2. 2 × P35S::HPT::T35STnos). This same approach was also used for pC-qsh1-T23 construct preparation.

For pC-qsh1-T1 vector preparation, the chimeric primers containing the qsh1-T1 target site sequence were cloned into the pYLsgRNA-U3 sgRNA cassette (Ma et al., 2015). Synthesis of the gRNA expression cassette was achieved using the U-F/gRNA-R primers (Supplementary Table S1) for an initial round of overlapping PCR, followed by a second round using the site-specific B1’/BL primers (Supplementary Table S1). The qsh1-T1-sgRNA constructs were then cloned into the pYLCRISPR/Cas9Pubi-H CRISPR/Cas9 Multi-targeting vector at a *Bsa*I site. The pC-qsh1-T1 vector was prepared via ligation of qsh1-T1 sgRNA expression cassettes into the pYLCRISPR/Cas9Pubi-H vector. Primers used for PCR reactions are compiled in Supplementary Table S1.

### Rice Transformation

The pC-qsh1-Tab or pC-qsh1-T1 or pC-qsh1-T23 binary vectors were introduced into the *Agrobacterium* tumefaciens EHA105. The parental *indica* hybrid rice varieties with stronger seed shattering (*Oryza satva* L. cv. HR1128 and Guangzan63-4S) were then transformed either as descried previously (Hiei et al., 2014) or by Wuhan Biorun biological technology Co., Ltd. After 4 weeks of rooting, regenerated plants were then transferred to plastic buckets and were grown in a greenhouse at 28°C/26°C (during the day and night, respectively).

### Mutant Plants Identification and Characterization

In order to confirm the successful targeted mutagenesis of rice plants, genomic DNA (gDNA) was collected via the cetyltrimethyl ammonium bromide (CTAB) method from a minimum of five leaves from different tillers during the mature period of rice growth (Rowland and Nguyen, 1993). This isolated gDNA then served as a template for PCR amplification with KOD FX (TOYOBO, Osaka, Japan). Specific primers were then used to amplify the genomic regions that contained the CRISPR/Cas9 target sites (Supplementary Table S1), after which these PCR fragment were purified and subjected to sequencing. Transgenic plant sequences were compared to those of wild-type (WT) plants in order to identify any mutations located therein. When mutations were associated with a normal sequencing chromatogram, plants were considered to be homozygous. In contrast, mutations that exhibited superimposed sequencing chromatograms were considered to be heterozygous or biallelic. Samples with such superimposed sequence chromatograms were cloned into the pEASY-Blut vector (TransGen Biotech, Beijing, China) and 10 positive clones underwent sequencing as a means of determining the mutation genotype. The DNAMAN5.0 and MAGE4.0 software were used for sequence alignment analysis.

### Analysis of Transgene-Free Plants

Plants of the T1 generation were used to identify transgene-free plants. For this analysis, *HPT-* and *Cas9*-specific PCR primers (Supplementary Table S1) and agarose gel electrophoresis were used to analyze transgenic plants, with pYLCRISPR/Cas9Pubi-H plasmids and WT plants serving as positive and negative controls, respectively. Plants that were negative for *HPT-* and *Cas9*- were considered to be transgene-free.

### Analysis of Seed Shattering Phenotype

The propensity of individual lines to undergo seed shattering was analyzed by respective collecting three panicles from different main tillers of each plant once they were mature. A digital force gauge was then used to measure the breaking tensile strength (BTS) at which grains were detached from the pedicle when they were pulled by hand (Qin et al., 2010). This test was repeated for a total of five grains from the uppermost part of each panicle per plant, with three biological replicates per line being analyzed in this manner.

### *qSH1* Expression and qSH1 Amino Acid Sequence Analyses

Total RNA was isolated from panicles of HR1128-WT and transgene-free homozygous mutant plants using the RNAprep pure Plant Kit (TIANGEN, Beijing, China). Total RNA (1 μg) was reverse-transcribed by using One-Step gDNA Removal and cDNA Synthesis SuperMix (TRNSGEN, Beijing, China). qRT-PCR was performed with gene-specific primers (Supplementary Table S1) using TB Green Premix Ex Taq Ta (TAKARA, Kusatsu, Japan) and Roche LightCycler*480* system. Ubiquitin was used an internal standard. Then the amino acid sequence of qSH1 was deduced and aligned for HR1128-WT and transgene-free homozygous mutant plants by using the MAGE4.0 and DNAMAN5.0 software.

### Histological Analysis

At 40 days post-heading, panicles of HR1128-WT and transgene-free homozygous mutant plants were harvested for abscission zone (AZ) observation using an approach previously detailed by Ji et al. (2016), with samples being analyzed via light microscopy following Fast Green FCF and Safranine staining.

### Agronomic Trait Characterization

In order to assess the major agronomic traits of transgene-free homozygous mutant lines, wild-type, promising hybrid rice lines, and control-check, these plants were grown under standard conditions in fields located in Changsha or Sanya. Traits including plant height, number of productive panicles, number of grains per panicle, seed setting rates, the weight of 1,000 seeds, and the length of the mature panicle were all measured in three biological replicates per line.

## Results

### Site-Directed Mutagenesis of *qSH1*

In an effort to efficiently and specifically mutate the *qSH1* gene in rice, we prepared three different CRISPR/Cas9 vectors including two containing two target sgRNAs (pC-qsh1-Tab and pC-qsh1-T23) and one containing a single-target sgRNA (pC-qsh1-T1). Both target-sites a and b (Figure 1A) contained the initiation codon and open reading frame of *qSH1*, while targets 1, 2, and 3 (Figure 1A) were all located near to a key SNP in the *qSH1*’s 5′ regulatory region that is associated with reduced seed shattering (Konishi et al., 2006). We then prepared three binary vectors (pC-qsh1-Tab, T1, T23; Figure 1B) based upon a previously described CRISPR/Cas9 vector (Ma et al., 2015). These three vectors were then introduced into the HR1128 and GZ63-4S rice varieties via *Agrobacterium*-mediated transformation. After transformation, site-specific PCR and Sanger sequencing were used to identify 7, 6, and 10 HR1128 mutants that had been recovered from 11, 7, 13 T0 transgenic HR1128 plants transformed using the pC-qsh1-Tab (63.64%, 7/11), T1 (85.71%, 6/7), and T23 (76.92%, 10/13) vectors, respectively. In addition, 6 GZ63-4S mutants were recovered from 12 T0 transgenic GZ63-4S plants that had undergone transformation with the pC-qsh1-Tab construct (50.00%, 6/12). These results revealed that these pC-qsh1-Tab, T1, and T23 constructs were all capable of rapidly generating *qsh1*-mutant rice plants.

### Characterization of *qSH1* Mutant Plants

**FIGURE 2:**
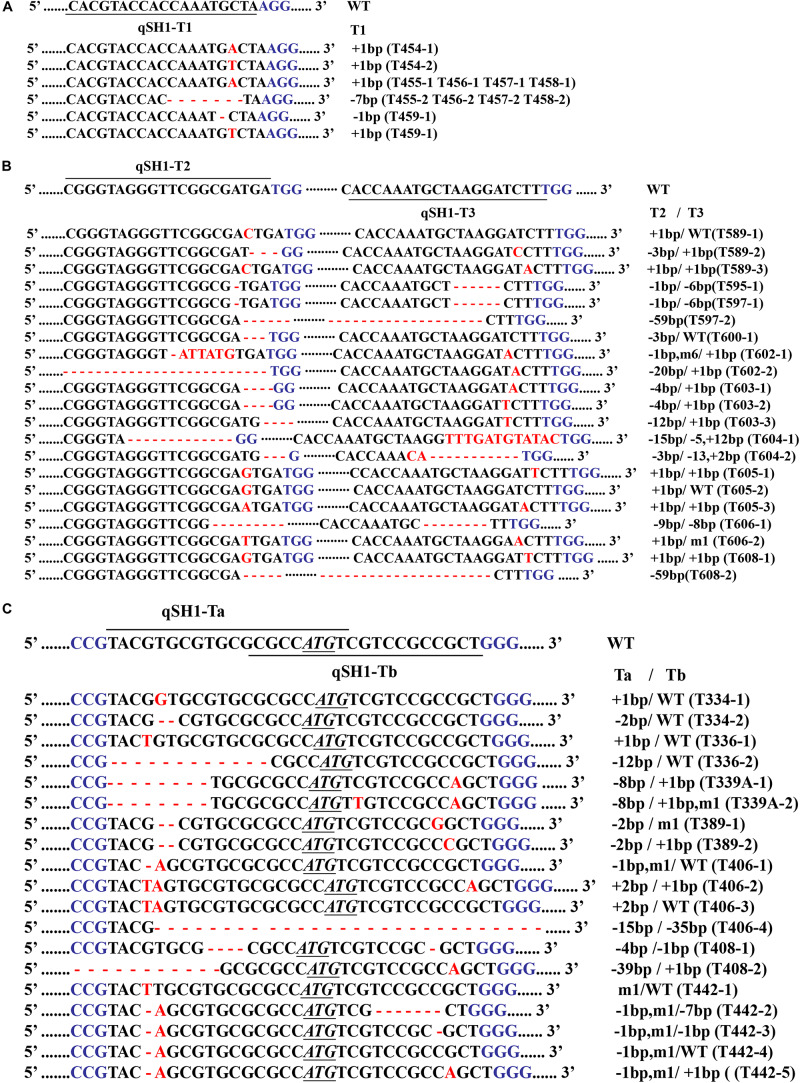
**(A)** is the mutant genotypes of pC-qsh1-T1 HR1128, **(B)** is the mutant genotypes of pC-qsh1-T23 HR1128, and **(C)** is the mutant genotypes of pC-qsh1-Tab HR1128. CRISPR/Cas9-induced *qSH1* gene modification in rice. Nucleotide sequences at the target site in the 6, 10, and 7 T0 mutant rice plants. The recovered mutated alleles are shown below the wild-type sequence. The target site nucleotides are indicated using black lines. The PAM site is highlighted in blue. Insertions are represented using red letter, while deletions are shown using red hyphens. The initiation cordon is shown in black as an underlined and italicized sequence. WT corresponds to wild-type. The numbers on the right indicate the type of mutation and the number of nucleotides involved. “–,” “ +, ” “m” indicate the deletion, insertion, and substitution of the indicated number of nucleotides, respectively.

**FIGURE 3:**
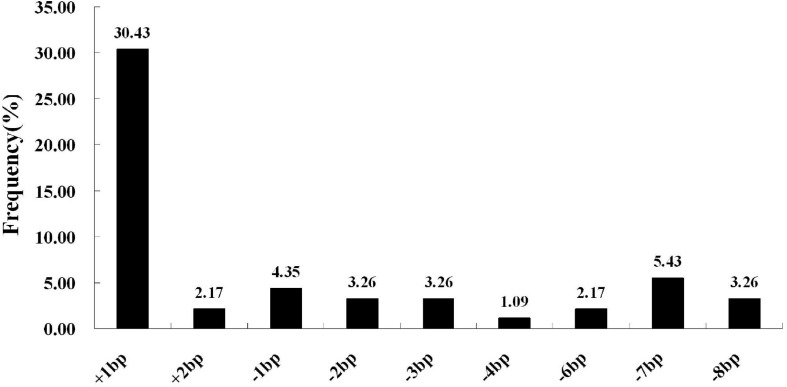
CRISPR/Cas9-induced mutation types and frequencies. The horizontal axis corresponds to different mutation types, whereas the vertical axis corresponds to the frequency at which each type was detected. Of all induced mutation types, single-nucleotide insertions were most frequently detected.

We next selected 7 (pC-qsh1-Tab, HR1128), 6 (pC-qsh1-T1, HR1128), and 10 (pC-qsh1-T23, HR1128) T0 *qsh1*-mutant plants that were then used to analyze the target site mutation characterization following transformation. For this approach, we cloned the target-PCR fragments into the pEASY-Blut vector after which positive clones were sequenced. We were then able to classify mutant genotypes into three categories (Table 1 and Figure 2): (1) homozygotes (20.00 to 28.57% mutation efficiency; example: T339A at target-site a); (2) bi-allelic (42.86 to 100.00% mutation efficiency; example: T334 at target-site a); and (3) chimeras (10.00 to 28.57% mutation efficiency; example: T406 at target-site a). The most commonly detected mutations were bi-allelic, in line with previous studies (Zhang et al., 2014; Ma et al., 2015; Tang et al., 2017). Six different types of mutations were identified, including insertions, deletions, substitutions, insertions + substitutions, deletions + substitutions, and insertions + deletions (Table 1). The frequencies of insertions, deletions, and substitutions ranged from 21.05 to 58.33%, 21.05 to 73.68%, and 4.76 to 5.27%, respectively. Single nucleotide insertions were the most common form of mutation, accounting for 30.43% of all such mutations (Figure 3).

**TABLE 1:** Ratios of mutant genotype and mutation type at the different target sites in T0 mutant plants.

Target site	Host cultivar	No. of examined plants	Mutation genotype ratios (%)^*a*^					Mutation type ratios (%)^*b*^						
			Homozygote ratios	Heterozygote ratios	Bi-allele ratios	Chimera ratios	Wild-type	Insertion	Deletion	Substitution	In + Sub	De + Sub	De + In	Wild-type
Target-a	HR1128	7	28.57 (2/7)	–	57.14 (4/7)	14.29 (1/7)	–	21.05 (4/19)	73.68 (14/19)	5.27 (1/19)	–	–	–	–
Target-b	HR1128	7	–	–	42.86 (3/7)	28.57 (2/7)	28.57 (2/7)	26.31 (5/19)	21.05 (4/19)	5.27 (1/19)	5.27 (1/19)	–	–	42.10 (8/19)
Target-1	HR1128	6	–	–	100.00 (6/6)	–	–	58.33 (7/12)	41.67 (5/12)	–	–	–	–	–
Target-2	HR1128	10	20.00 (2/10)	–	80.00 (8/10)	–	–	33.33 (7/21)	61.90 (13/21)	–	–	4.77 (1/21)	–	–
Target-3	HR1128	10	20.00 (2/10)	–	60.00 (6/10)	10.00 (1/10)	10.00 (1/10)	47.62 (10/21)	23.81 (5/21)	4.76 (1/21)	–	–	9.52 (2/21)	14.29 (3/21)

**^*a*^Based on the number of each mutant genotype out of the total number of all mutant genotypes at different the target sites. ^*b*^Based on the number of each allele mutation type out of the total number of all allele mutation types at different the target sites.**

While powerful, the CRISPR/Cas9 technology is susceptible to the introduction of off-target mutations, as reported previously (Endo et al., 2014; Puchta, 2016). We therefore assessed the off-target efficiency of qSH1-Ta, qSH1-Tb, and qSH1-T1 at sites which have high sequence similarity (<5 mismatch bp) to our target sites. We examined four different potential off-target sites within the rice genome, but we detected no evidence of off-target events among 40 T1 plants (Supplementary Table S2). As such, our findings suggest that the sgRNAs used herein were able to achieve high mutagenic specificity.

### Development of Transgene-Free Mutant T1 Rice Plants

**FIGURE 4:**
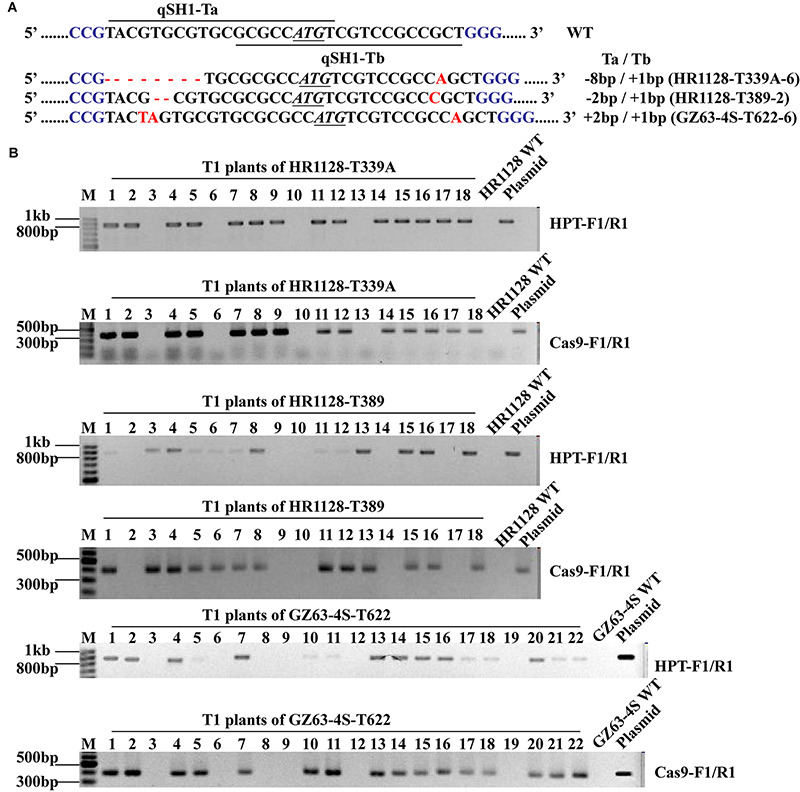
Mutation types and PCR-based identification of transgene-free in the T1 generation. **(A)** The mutation types of HR1128-T339A-6, HR1128-T389-2, and GZ63-4S-T622-6 **(B)**. *HPT* and *Cas9* gene-specific PCR amplification of HR1128-T339A, HR1128-T389, and GZ63-4S-T622 T1 generation plants.

We next sought to develop rice plants containing homozygous *qSH1* mutations that were free of any transferred DNA (T-DNA) derived from the pC-qsh1-Tab, T1, or T23 constructs. In order to test for such plants, we utilized *HPT-* and *Cas9*-specific PCR primers (Supplementary Table S1) to amplify DNA from T1 populations derived from five T_0_ lines. T-DNA-free plants were those in which neither *Cas9* nor *HPT* were detectable, and we were able to identify such plants at frequencies of 21.43 to 27.78% among analyzed plants (Supplementary Table S3 and Figure 4). This confirmed that T1 rice plants could be utilized to effectively develop transgene-free rice plants, as the pYLCRISPR/Cas9 vector and the *qSH1* mutations are inherited independently of one another. We then selected three transgene-free plants bearing homozygous *qSH1* mutations including plants with coding frameshifts, premature translational stops and altered 5′-UTR regions (HR1128-T339A-6, HR1128-T389-2, GZ63-4S-T622-6), and we then used these plants to generate the T2 population (Figure 4).

### T2 *qSH1* Mutant Lines Exhibit Significantly Reduced Seed Shattering Without any Significant Changes in Major Agronomic Traits

**FIGURE 5:**
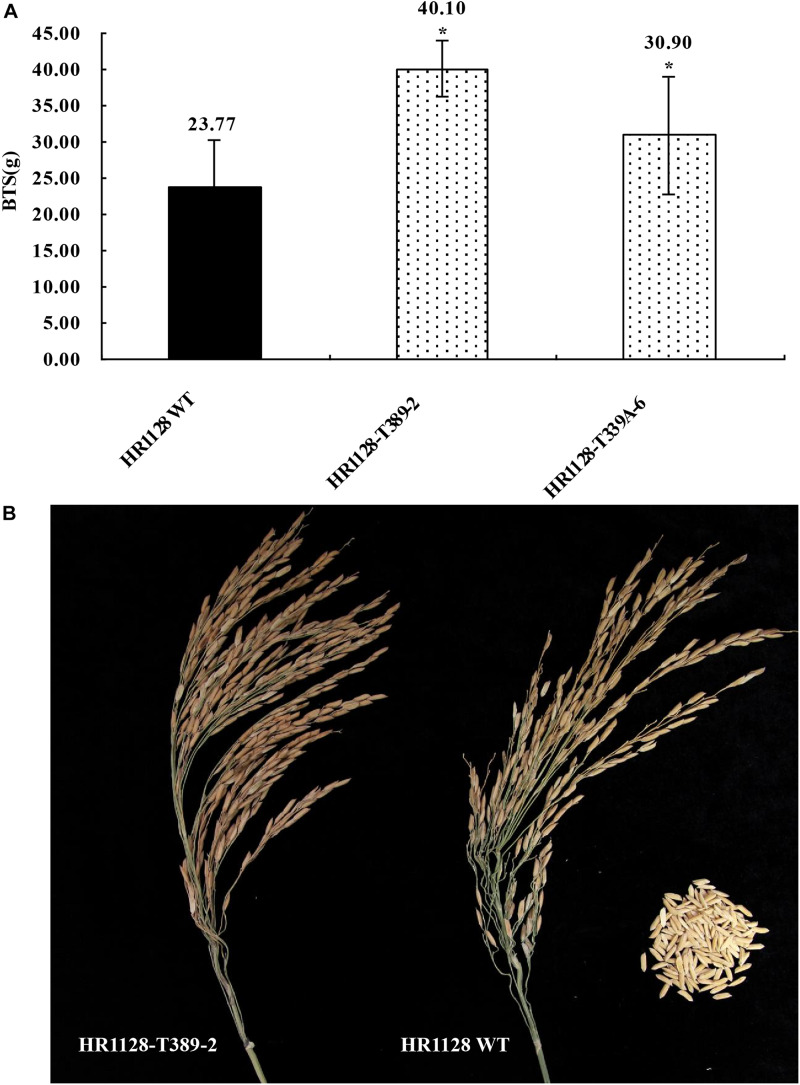
Characterization of seed shattering phenotypes in *qsh1* transgene-free homozygous mutant lines. **(A)** The breaking tensile strength of *qsh1* mutant lines (HR1128-T389-2, HR1128-T339A-6 mutant lines) and HR1128-WT line. *P < 0.05. **(B)** The seed shattering phenotype of *qsh1* mutant lines (HR1128-T389-2 mutant lines) and HR1128-WT line.

We next measured the breaking tensile strength (BTS) of two T2 transgene-free homozygous mutant lines (HR1128-T339A-6, HR1128-T389-2) as a means of gauging the seed shattering behavior of these mutants relative to that of HR1128-WT. In this analysis, BTS values was negatively correlated with degree of shattering. We were able to detect significant differences in pulling strength when comparing HR1128-WT and mutant plant lines (*P* < 0.05; Figure 5). Specifically, we found that *qSH1* mutants exhibited significantly higher BTS values relative to HR1128-WT, indicating that our CRISPR/Cas9-mediated editing of the *qSH1* gene was able to significantly reduce the seed shattering of rice.

In order to establish whether these *qsh1* mutations had any impact on the major agronomic traits in mutant lines, we next analyzed both the HR1128-WT and the two transgene-free T2 mutant lines derived therefrom. When we measured plant height, number of productive panicles, number of grains per panicle, seed setting rate, weight of 1,000 seeds, and panicle length, we observed no significant differences in any of these traits between HR1128-WT and mutants under normal growth conditions (Table 2). As such, these findings indicate that the targeted mutagenesis of *qSH1* has no impact on major agronomic traits when mutants are cultivated under standard conditions.

**TABLE 2:** Agronomic traits of two transgene-free homozygous T2 mutant lines and HR1128-WT plants.

Lines	Plant height (cm)	The number of productive panicles	The number of grains per panicle	The seed setting rate (%)	1,000 seed weight (g)	The length of panicle (cm)
HR1128-WT	133.73 ± 5.02^*a*^	4.00 ± 0.00^*a*^	455.33 ± 66.16^*a*^	75.40 ± 8.12^*a*^	26.00 ± 0.55^*a*^	29.00 ± 2.00^*a*^
HR1128-T339A-6	129.50 ± 1.50^*a*^	4.33 ± 0.58^*a*^	419.33 ± 30.27^*a*^	70.41 ± 5.50^*a*^	25.21 ± 1.52^*a*^	28.33 ± 1.53^*a*^
HR1128-T389-2	130.67 ± 1.26^*a*^	4.67 ± 0.58^*a*^	467.33 ± 37.00^*a*^	70.65 ± 6.99^*a*^	23.83 ± 1.71^*a*^	28.83 ± 1.89^*a*^

**The results are shown for five plants of each mutant line and are represented as the mean ± SE. The values marked with the same letter (a) are non-significantly different (P < 0.05, Student’s t-test).**

### Analysis of the *qSH1* Expression and qSH1 Amino Acid Sequence in Transgene-Free Homozygous Mutant Plants

**FIGURE 6:**
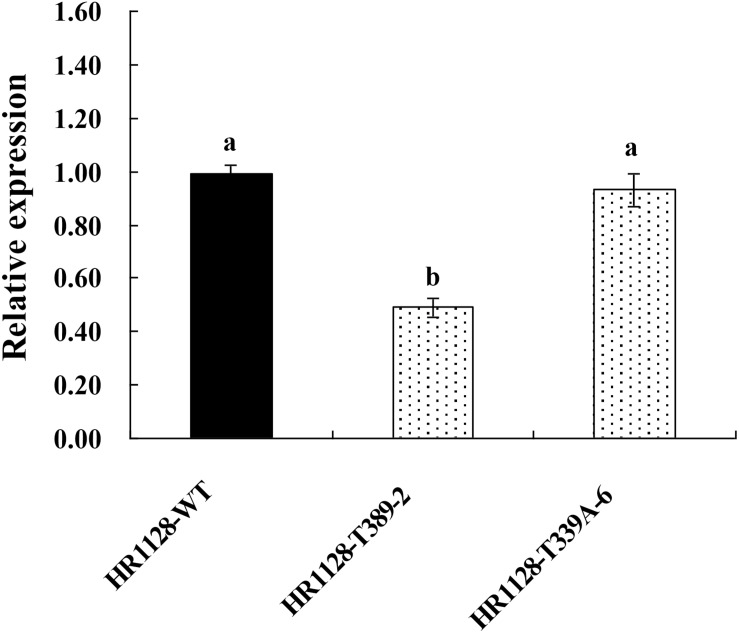
The expression of *qSH1* gene in WT and mutant lines. qRT-PCR analysis of the transcription of *qSH1* gene in WT line (HR1128-WT) and mutant lines (HR1128-T389-2, HR1128-T339A-6).

**FIGURE 7:**
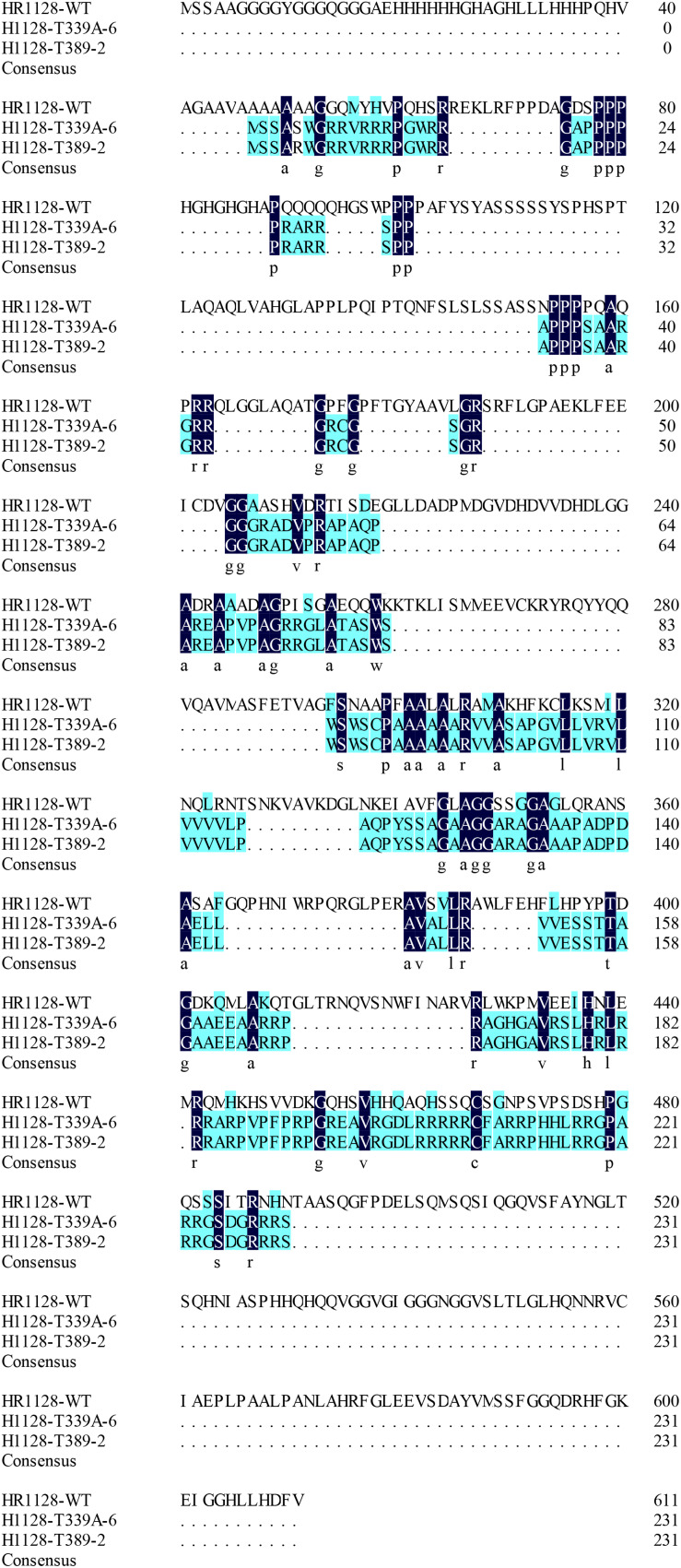
The amino aid sequence of qsh1 transgene-free homozygous mutant lines. Deduced qSH1 amino aid sequence alignment for the two transgene-free homozygous mutant lines (HR1128-T389-2, HR1128-T339A-6) and for WT line (HR1128-WT). Each of the mutant alleles codes for truncated and disrupted qSH1 proteins.

We measured the relative expression of *qSH1* in HR1128-WT and transgene-free homozygous mutant plants by quantitative real-time polymerase chain reaction (qRT-PCR) and found the expression of *qSH1* was significantly reduced in HR1128-T389-2 and non-significantly different in HR1128-T339A-6, compared with the HR1128-WT (Figure 6). Next we deduced and aligned the qSH1 amino acid sequences from the HR1128-T339A-6, HR1128-T389-2, and HR1128-WT plants, revealing that each of the two mutant plants encoded the qSH1 protein that was only 231 amino acids in length, whereas the HR1128-WT qSH1 protein was 611 amino acids long (Figure 7). The amino acid sequences in these mutant proteins were also altered, emphasizing that these mutant alleles expressed truncated, disrupted and altered qSH1 proteins.

### Analysis of the Abscission Zone in Mutant Lines

**FIGURE 8:**
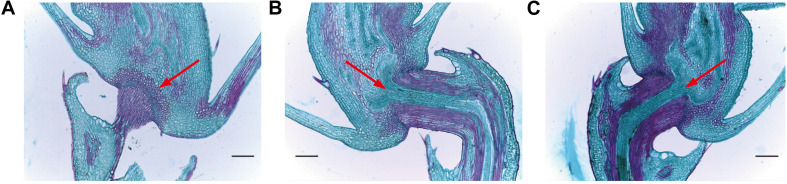
Longitudinal sections of the abscission zone in grain pedicle tissues. **(A)** Longitudinal abscission zone (AZ) sections from HR1128-WT. **(B)** Longitudinal abscission zone (AZ) sections from HR1128-T389-2. **(C)** Longitudinal abscission zone (AZ) sections from HR1128-T339A-6. Magnification time: ×100. Arrows indicate the AZ. Scale bar: 100 μm.

In order to assess abscission zone (AZ) morphology of our mutant lines, we next analyzed them via optical microscopy (Figure 8). We observed that the AZ between the pedicle and the spikelet at the rice seed base in these mutant lines was partially- developed (Figure 8), with a weaker seed shattering phenotype than that of HR1128-WT. In contrast, the AZ of HR1128-WT was well-developed. This indicates that the targeted mutagenesis of *qSH1* resulted in a partial disruption of AZ formation, leading to the improved “easy-to-shatter” phenotype observed in these mutants.

### Development of New Promising Hybrid Rice Lines With Reduced Seed Shattering

**FIGURE 9:**
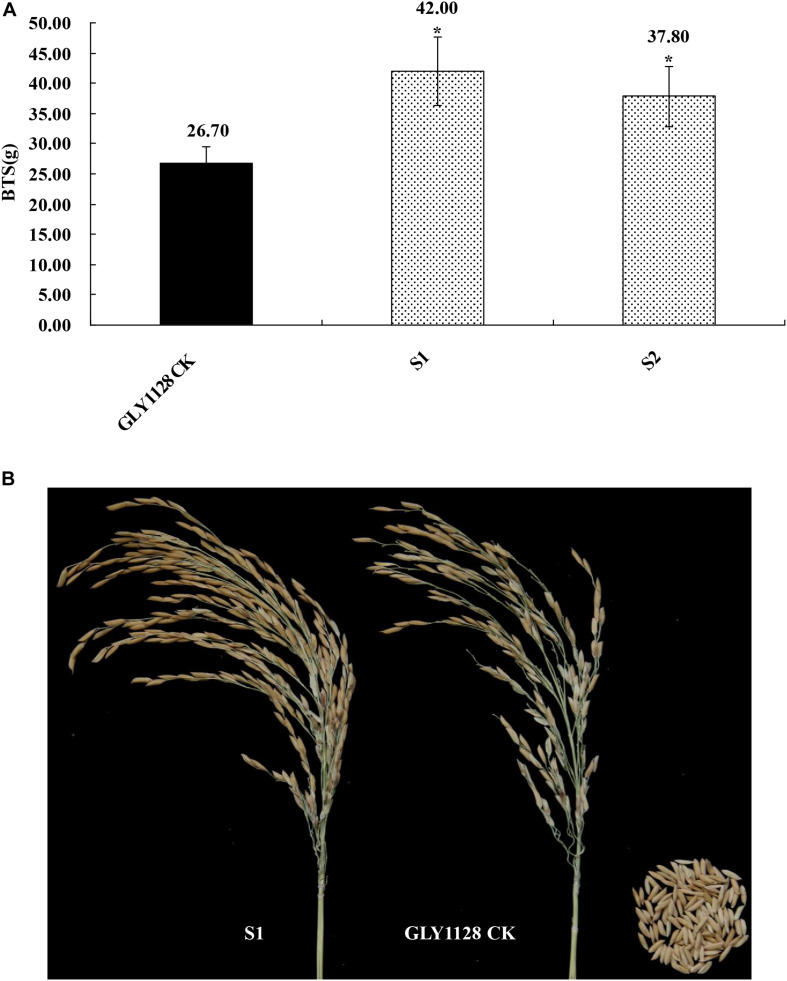
Characterization of the seed shattering phenotype in promising hybrid rice lines. **(A)** The breaking tensile strength of promising hybrid rice lines (S1, S2) and GLY1128 CK. *P < 0.05. **(B)** The seed shattering phenotype of the promising hybrid rice line (S1) and GLY1128 CK.

We next crossed the HR1128 mutants (HR1128-T339A-6, HR1128-T389-2) with the GZ63-4S mutant (GZ63-4S-T622-6) in order to generate two independent mutant hybrid rice lines (S1, S2). As a control, we additionally chose the commercial hybrid rice GLY1128 (by crossing HR1128-WT and GZ63-4S-WT). We then grew these mutant hybrid rice lines and GLY1128 line (CK) under normal conditions. When we analyzed the BTS values for these rice lines, we found that the S1 and S2 had significantly greater pulling strength values relative to the CK line (Figure 9), suggesting that the mutation of both parental lines was sufficient to improve the seed shattering of the resultant hybrid line (Table 3). Importantly, these new promising hybrid rice lines did not exhibit any significant morphological differences or changes in grain yield relative to control line (Table 3). These results together clearly demonstrate that CRISPR/Cas9-mediated mutagenesis of the *qSH1* gene can be used to generate new promising hybrid rice lines with an intermediate seed shattering phenotype as a means of reducing yield losses during mechanized rice harvesting.

**TABLE 3:** Agronomic traits of two promising hybrid lines and GLY1128-CK plants.

Lines	Plant height (cm)	The number of productive panicles	The number of grains per panicle	The seed setting rate (%)	1,000 seed weight (g)	The length of panicle (cm)
GLY1128-CK	158.33 ± 1.53^a^	7.00 ± 0.00^a^	429.00 ± 16.37^a^	73.06 ± 1.16^a^	26.73 ± 1.06^a^	27.37 ± 1.10^a^
S1	158.00 ± 2.00^a^	7.33 ± 0.58^a^	447.00 ± 47.00^a^	68.44 ± 3.03^a^	26.03 ± 1.66^a^	27.53 ± 0.74^a^
S2	159.00 ± 2.00^a^	7.67 ± 0.58^a^	426.67 ± 33.50^a^	73.10 ± 6.03^a^	26.60 ± 0.58^a^	27.77 ± 0.65^a^

**The results are shown for five plants of each mutant line and are represented as the mean ± SE. The values marked with the same letter (a) are non-significantly different (P < 0.05, Student’s t-test).**

## Discussion

In domesticated, rice seed shattering is disadvantageous as it results in significant losses during harvesting, leading to efforts to develop crop varieties with reduced seed shattering (Doebley, 2006). Despite these efforts, many rice varieties are still exhibit the undesirable “easy-to-shatter” phenotype, with this trait being particularly pronounced among *indica* subspecies. The *qSH1* gene has been identified as a key regulator of seed shattering trait, with loss of expression of this gene at the mRNA level having been linked to reduced seed shattering. Meanwhile *qSH1* functions downstream of *SHAT1* and *SH4* so as to maintain their expression in the AZ, leading to the promotion of AZ differentiation and showing stronger seed shattering (Konishi et al., 2006; Zhou et al., 2012). Previous efforts to develop intermediate seed shattering rice varieties have largely relied upon γ irradiation followed by crossing, backcrossing and marker assisted selection (MAS) during breeding, with no prior reports of the development of intermediate-shatter mutants produced via targeted genetic editing. More recently, CRISPR/Cas9-mediated gene editing has been used to genetically modify rice (Zhou et al., 2016; Wang et al., 2016; Tang et al., 2017; Zhang et al., 2019), maize (Feng et al., 2016), wheat (Puchta, 2016), and other crops. Few studies to date, however, have used this approach to edit the parental lines that are used to produce promising hybrid rice varieties. HR1128 is an elite *indica* restorer line with a lodging resistance, high seed setting rate and large panicle that has been used to generate many different hybrid rice varieties including GuangLiangYou1128 (GLY1128), LiangYou1128 (LY1128), and YLiangYou1128 (YLY1128). HR1128 and its derivatives lines, however, exhibit undesirably strong seed shattering phenotype. In an effort to overcome this limitation, we employed a CRISPR/Cas9 approach to knock-out the *qSH1* gene in the parental lines of GLY1128, as the CRISPR/Cas9 technology allows for highly efficient mutagenesis without any sustained transgene insertion in progeny plants.

In the present study, we produced three transgene-free homozygous mutant lines (HR1128-T339A-6, HR1128-T389-2, GZ63-4S-T622-6) in the T2 generation. By further analyzing the *qSH1* expression, qSH1 amino acid sequence and assessing the AZ of HR1128-T339A-6, HR1128-T389-2, and HR1128-WT lines, we found that qSH1 proteins were truncated, altered, and disrupted, and that AZ formation was partially disrupted in these mutants, and the HR1128-T389-2 showed significantly reduced *qSH1* expression. Previous studies showed that mutations in *cis*-regulatory regions/elements often cause a change in the expression level and/or pattern of the genes (Li et al., 2017), thus the deletions in 5′-UTR region of HR1128-T389-2 may lead to *qSH1* expression significantly reduced. Meanwhile, 1bp insertion in CDS possible activate the nonsense-mediated mRNA decay (NMD) pathway and the process of callus differentiation during transgenic tissue culture also may cause transposon activation, etc. can affect the mRNAs stability (Yin et al., 2007; Christopher and Rachel, 2012), as a result genes expression level of mutants have changed. In summary, we speculated that a loss-of-function of the qSH1 protein was a primary cause of incomplete AZ formation in these mutant plants, resulted in their improved strong seed shattering phenotype and reduced expression of *qSH1* may promote for this. Then we produced two promising hybrid rice lines (S1, S2) with an intermediate seed shattering phenotype by crossing the HR1128 mutant line and the GZ63-4S mutant line. These S1 and S2 lines exhibited favorable agronomic traits, were transgene-free, and could be readily used for farming production if permitted by governmental policies. Overall, our results highlight the potential for the use of CRISPR/Cas9 technology to develop promising hybrid rice lines and parental varieties bearing targeted mutations capable of improving the “easy-to-shatter” trait, with *qSH1* being a potentially ideal target for the accelerated development of intermediate seed shattering rice varieties.

Heterosis utilization has greatly benefited crop breeding efforts, and recent changes in rice breeding practices have significantly improved rice grain yields owing to the efficient use of heterosis (Wang et al., 2005; Cheng et al., 2007). In hybrid rice varieties (*Oryza sativa*), heterozygous first filial (F_1_) generation plants typically exhibit a 10–20% yield advantage over their parental lines (Cheng et al., 2007; Li et al., 2007; Luo et al., 2013), making heterosis an ideal property to leverage for rice breeding efforts. More recently, CRISPR/Cas9 technology has been developed and widely employed as a means of improving a range of crops. However, few studies to date, have combined CRISPR/Cas9 approach with heterosis utilization to produce new varieties, for example via editing the parental lines that are used to produce promising hybrid rice varieties. In the present study, we combined heterosis utilization with targeted gene editing as a means of rapidly producing new promising hybrid rice lines with an improved “easy-to-shatter” phenotype and beared desirable agronomic traits, suggesting that integrating both CRISPR/Cas9 approach and heterosis utilization may potentially represent a powerful, highly efficient, and green approach to the genetic improvement of rice and other hybrid crops breeding. At present the large panicle and large grain size traits are major reasons for high-yield in hybrid rice, however, these favorable agronomic traits easily lead to “easy-to-shatter” and cause serious yield losses. Therefore, reducing strong seed shattering being one beneficial approach to the achieving high rice yields, as a result this study offer a novel strategy and new materials for breeding new rice varieties with an intermediate seed shattering and desirable agronomic traits via combining CRISPR/Cas9 technology with heterosis utilization.

## Data Availability Statement

All datasets generated for this study are included in the article/Supplementary Material.

## Author Contributions

DyY and MD designed the research work and drafted the manuscript. XS performed the experiments, analyzed the data, and drafted the manuscript. ZS and XW modified the drafted. YT, DoY, and GY participated in performing the experiments.

## Conflict of Interest

The authors declare that the research was conducted in the absence of any commercial or financial relationships that could be construed as a potential conflict of interest.

## Footnotes

1 http://skl.scau.edu.cn/

2 http://blast.ncbi.nlm.nih.gov/Blast.cgi
