# Supplementary material for: Improvement of the Rice “Easy-to-Shatter” Trait via CRISPR/Cas9-Mediated Mutagenesis of the qSH1 Gene
Source: Front Plant Sci. 2020 May 25;11:619. doi: 10.3389/fpls.2020.00619 (PMC7262966; doi:10.3389/fpls.2020.00619)
Supplement: Supplementary file 1 [file Table_1.DOC]

**Supplementary Materials**

**Supplementary Table 1. Primers used in this study**

| Primer name | Base sequence (5’→3’) | Purpose |
| --- | --- | --- |
| Ta-F | GGCACATGGCGCGCACGCACGTA | Construction of qSH1-Ta target site |
| Ta-R | AAACTACGTGCGTGCGCGCCATG | Construction of qSH1-Ta target site |
| Tb-F | GCCGCGCCATGTCGTCCGCCGCT | Construction of qSH1-Tb target site |
| Tb-R | GGCACATGGCGCGCACGCACGTA | Construction of qSH1-Tb target site |
| T1-F | GGCACACGTACCACCAAATGCTA | Construction of qSH1-T1 target site |
| T1-R | AAACTAGCATTTGGTGGTACGTG | Construction of qSH1-T1 target site |
| T2-F | GCCACGGGTAGGGTTCGGCGATGA | Construction of qSH1-T2 target site |
| T2-R | AAACTCATCGCCGAACCCTACCCG | Construction of qSH1-T2 target site |
| T3-F | GCCGCACCAAATGCTAAGGATCTT | Construction of qSH1-T3 target site |
| T3-R | AAACAAGATCCTTAGCATTTGGTG | Construction of qSH1-T3 target site |
| U-F | AAACTACGTGCGTGCGCGCCATG | U#-T-gRNA expression-box frist PCR |
| gRNA-R | GCCGCGCCATGTCGTCCGCCGCT | U#-T-gRNA expression-box frist PCR |
| B1’ | TTCAGAGGTCTCTCTCGCACTGGAATCGGCAGCAAAGG | U#-T-gRNA expression-box second PCR |
| B2 | AGCGTGGGTCTCGTCAGGGTCCATCCACTCCAAGCTC | U#-T-gRNA expression-box second PCR |
| B2’ | TTCAGAGGTCTCTCTGACACTGGAATCGGCAGCAAAGG | U#-T-gRNA expression-box second PCR |
| BL | AGCGTGGGTCTCGACCGGGTCCATCCACTCCAAGCTC | U#-T-gRNA expression-box second PCR |
| SP1 | CCCGACATAGATGCAATAACTTC | CRISPR/Cas9 expression-vector verification |
| SP2 | GCGCGGTGTCATCTATGTTACT | CRISPR/Cas9 expression-vector verification |
| HPT-F1 | CTTCTGCGGGCGATTTGT | Transgenic plant detection |
| HPT-R1 | CAGCGTCTCCGACCTGAT | Transgenic plant detection |
| Cas9-F1 | GTTGGTATTCACGGGGTGCCT | Transgenic plant detection |
| Cas9-R1 | CGACGATGTTGCCGAAGATGG | Transgenic plant detection |
| qSH1-JC-F1 | AGTTTCACGGCTTACCATTTCC | Ta,Tb target mutation detection |
| qSH1-JC-R1 | ACCATGAGGCGGTGGCGAGT | Ta,Tb target mutation detection |
| qSH1-JC-F2 | TTTTCTTTTCCTCTGCTCGACTC | T1,T2,T3 target mutation detection |
| qSH1-JC-R2 | ATCATGTATTTTAAGGGGCACGA | T1,T2,T3 target mutation detection |
| qSH1-Q-F | GTGCTTGAAGAGCATGATTCTG | T1 expression of *qSH1* detection |
| qSH1-Q-R | CTAAACAAGGAGATCGCCGT | T1 expression of *qSH1* detection |
| UbQ5-Q-F | ACCACTTCGACCGCCACTACT | internal standard of qRT-PCR |
| UbQ5-Q-R | ACGCCTAAGCCTGCTGGTT | internal standard of qRT-PCR |

**Supplementary Table 2. Mutation detections in the putative CRISPR/Cas9 off-target sites.**

| Target sit | Putative off-target sit | Putative off-target locus | Sequence of putative off-target site^a^ | No. of mismatching bases | No. of planes detected | No. of plants with mutations |
| --- | --- | --- | --- | --- | --- | --- |
| qSH1-T1 | off-T1-1 | Chr[3: 32619247-32619266](http://ensembl.gramene.org/Oryza_indica/Location/View?r=3:32619245-32619270;tl=IpXcffa7e8tXfoAc-14772-4911158) | GCTTGTACCACCAAATGCAATGG | 3 | 20 | 0 |
| qSH1-T2 | / | / | / | / | / | / |
| qSH1-T3 | / | / | / | / | / | / |
| qSH1-Ta | off-Ta-1 | Chr1: 22254602-22254621 | CTCGGGCGCGCACGCACGTGGGG | 5 | 20 | 0 |
| qSH1-Tb | off-Tb-1 | Chr5: 23688474-23688493 | GCGCCATGTCGTCCGCCGCGGGG | 1 | 20 | 0 |
|  | off-Tb-2 | Chr4: 23232406-23232425 | CCTCCCGCTCGTCCGCCGCTGGG | 5 | 20 | 0 |

a.The base that matched the sgRNA are marked in red. The sequences of the PAM are underlined.

**Supplementary Table 3. CRISPR/Cas9-induced mutations in *qSH1* and Transgene-free ratio in T1 generation.**

| T0 plant | Target site | Host cultivar | No. of plants examined | Transgene-free ratio(%) in T1 |
| --- | --- | --- | --- | --- |
| T339A | qsh1-Tab | HR1128 | 18 | 22.22 (4/18) |
| T389 | qsh1-Tab | HR1128 | 18 | 27.78 (5/18) |
| T455 | qsh1-T1 | HR1128 | 12 | 0 (0/12) |
| T589 | qsh1-T23 | HR1128 | 14 | 21.43 (3/14) |
| T622 | qsh1-Tab | GZ63-4S | 22 | 27.27 (6/22) |
